# Theory of Mind and Executive Control Deficits in Typically Developing Adults and Adolescents with High Levels of Autism Traits

**DOI:** 10.1007/s10803-016-2735-3

**Published:** 2016-02-17

**Authors:** Elif Gökçen, Norah Frederickson, K. V. Petrides

**Affiliations:** London Psychometric Laboratory, University College London, 26 Bedford Way, London, WC1H 0AP UK; Clinical, Educational, and Health Psychology Research Department, University College London, London, UK

**Keywords:** Autism spectrum disorder, Subclinical autism traits, Theory of mind, Executive control, Alexithymia

## Abstract

Autism spectrum disorder (ASD) is characterised by profound difficulties in empathic processing and executive control. Whilst the links between these processes have been frequently investigated in populations with autism, few studies have examined them at the subclinical level. In addition, the contribution of alexithymia, a trait characterised by impaired interoceptive awareness and empathy, and elevated in those with ASD, is currently unclear. The present two-part study employed a comprehensive battery of tasks to examine these processes. Findings support the notion that executive function and theory of mind are related abilities. They also suggest that individuals with elevated levels of autism-like traits experience a partially similar pattern of social and executive function difficulties to those diagnosed with ASD, and that these impairments are not explained by co-occurring alexithymia.

## Introduction

Empathy is the ability to understand and share the affective experiences of others (Decety and Jackson 2006; Decety and Lamm 2006; Singer and Lamm 2009) and plays a pivotal role in the formation of successful human relationships (Baron-Cohen and Wheelwright 2004; Decety 2010; Dziobek et al. 2008; Rameson et al. 2012; Singer 2006). Two main components contribute to empathic processing: an affective component, which allows one to vicariously experience the feelings of others whilst understanding that they are distinct from one’s own, and a cognitive component (also referred to as metalizing, cognitive perspective-taking, or Theory of Mind; ToM), which involves the ability to understand and make inferences about what another person is thinking or feeling, without necessarily sharing that mental state (Frith and Frith 2003; Jones et al. 2010; Premack and Woodruff 1978; Schwenck et al. 2012; Shamay-Tsoory 2011; Shamay-Tsoory et al. 2009).

### Empathy and Autism Spectrum Disorder

Deficits in empathic functioning have frequently been cited as a core feature of autism spectrum disorder (ASD)—a lifelong neurodevelopmental condition marked by profound impairments in social interaction and communication, as well as repetitive behaviors and restricted interests (American Psychiatric Association 2013). Over the past few decades, a growing body of research has revealed ASD-related impairments in ToM. For instance, Baron-Cohen and Wheelwright (2004) documented lower levels of empathy for adults with ASDs using the empathy quotient (EQ), a 40-item self-report questionnaire that primarily focuses on the cognitive domain of empathy. Behavioral data from studies using static (e.g., Baron-Cohen et al. 1997, 2001a, c; Dziobek et al. 2006; Lahera et al. 2014), and more naturalistic video-based assessments of ToM (e.g., Dziobek et al. 2006; Heavey et al. 2000; Lahera et al. 2014; Ponnet et al. 2008) have also reported ASD-specific deficits in mental state attribution.

Conversely, results from studies examining affective empathy in ASD have been far less consistent. For instance, Minio-Paluello and colleagues (Minio-Paluello et al. 2009) found that individuals with ASD showed no sensorimotor resonance when observing another person in pain. Findings from related investigations in ASD samples, however, indicate typical physiological responses to others’ pain (Fan et al. 2014; Hadjikhani et al. 2014) and distress (Blair 1999). In fact, Smith (2009) suggests that autism is associated with heightened levels of affective empathy, and reports of greater responsiveness to others’ emotional states in children with ASD yield support for this hypothesis (Capps et al. 1993).

To date, only a small number of studies have jointly assessed the cognitive and affective components of empathy in ASD populations. For instance, using the Interpersonal Reactivity Index (Davis 1980), Rogers and colleagues (Rogers et al. 2007) reported reduced cognitive empathy in adults with Asperger syndrome, but found no impairments in empathic concern (a process related to affective empathy). A later study by Dziobek et al. (2008) also revealed dissociable deficits in empathic processing. These researchers found that whilst there were no group differences in the affective domain of the Multifaceted Empathy Test, individuals with ASD exhibited clear deficits in their ability to infer another person’s mental state. Studies investigating empathic processing in children with ASD also revealed cognitive empathy deficits. Findings from these studies suggest that while boys with ASD experience significant difficulties in mentalization, their capacity to resonate with another person’s emotional state remains intact (Jones et al. 2010; Schwenck et al. 2012). Taken together, these studies indicate that individuals with ASD show specific difficulties in mental state attribution, rather than a global deficit in empathic processing.

Recent examinations of ASD traits in the general population yield a similar pattern of results. For example, Gökçen et al. (2014) reported poorer ToM performance in typically developing adults with elevated levels of ASD traits. In a study investigating both domains of empathic functioning, higher ASD traits were associated with atypical perspective-taking abilities on the animated triangles task, but no impairments were found on a measure examining affective responses to emotional faces (Lockwood et al. 2013). In sum, these findings suggest that even in the absence of a clinical diagnosis, individuals with higher levels of autistic-like traits may be more susceptible than the general population to ASD-related empathy deficits.

### Alexithymia

An important consideration when examining empathy processes in ASD is the high comorbidity between autism and alexithymia. Alexithymia is a subclinical condition characterized by difficulties in the ability to recognize, express, and distinguish emotional states from bodily sensations (Nemiah et al. 1976). In recent years, studies have suggested that the affective and empathic deficits associated with autism may be a consequence of co-occurring alexithymia, rather than ASD *per se* (Bird et al. 2010; Cook et al. 2013; Silani et al. 2008), and that controlling for alexithymia reveals comparable levels of empathy in individuals with and without ASD (Bird and Cook 2013). Nevertheless, reports from studies examining autistic individuals (Fan et al. 2014) and ASD traits in typically developing populations (Lockwood et al. 2013) showed no significant effects of alexithymia on measures of empathic processing.

### Executive Function

A further consideration in the study of empathy is its relationship with executive function, which refers to a set of higher-order cognitive mechanisms facilitating adaptive and goal-directed behavior in a constantly changing environment (Corbett et al. 2009; Jurado and Rosselli 2007; Lezak 1995). Executive functions are thought to encompass several distinct, yet interrelated processes, such as planning, cognitive flexibility (or set-shifting), and response inhibition (Stuss and Knight 2002). To date, there has been a wealth of evidence suggesting a robust link between these higher-order processes and mentalizing ability. For instance, studies examining this association in typically developing children have found that better abilities in executive control were related to enhanced performance on ToM tasks, independent of intellectual functioning (Austin et al. 2014; Carlson et al. 2013; Carlson and Moses 2001; Carlson et al. 2002; Hughes 1998a, b; Sabbagh et al. 2006; though see also Pellicano 2007; Perner et al. 2002). Furthermore, growing empirical evidence suggests that the positive association between executive and ToM processes extends beyond childhood into adolescence and adulthood (Apperly et al. 2010; Bull et al. 2008; Dumontheil et al. 2010; Gökçen et al. 2014; Vetter et al. 2013).

Considerable attention has also been devoted to understanding the executive function and ToM link in autistic populations. Studies have shown that along with impaired mentalisation, individuals with autism (Dawson et al. 1998; McEvoy et al. 1993; Pellicano 2007; Robinson et al. 2009; for reviews see Hill 2004a, b; and Russo et al. 2007), or with elevated levels of ASD traits (Christ et al. 2010; Gökçen et al. 2014), exhibit significant deficits in multiple domains of executive processing. In addition, results from studies assessing both executive and ToM abilities in ASD have consistently revealed a link between the two constructs, independent of intellectual capacity (Joseph and Tager-Flusberg 2004; Ozonoff et al. 1991; Pellicano 2007). Similar results have also been obtained from a sample of neurotypical adults demonstrating higher and lower levels of ASD traits (Gökçen et al. 2014). Findings showed that adults in the high trait group displayed significantly poorer performance on tasks tapping ToM and cognitive flexibility, relative to their low trait counterparts. However, it is worth noting that other investigations have documented non-significant correlations between measures of ToM and executive functioning in individuals with ASD (Dziobek et al. 2006; Lahera et al. 2014).

Given the observed association and the coexistence of executive function and ToM deficits in autism, numerous studies have sought to establish the interrelationships between these cognitive domains in typical and atypical development. Whilst some researchers (Perner 1998, 2000; Perner and Lang 2000) contend that intact ToM fosters executive function, stronger support has emerged for the opposing view (Russell 1997, 2002), viz., that intact executive functioning is a prerequisite for ToM development in individuals with (Pellicano 2007) and without (Austin et al. 2014; Hughes 1998b; Hughes and Ensor 2007) ASD. Together, these findings highlight the importance of executive function skills in successful mentalization, and suggest that ToM impairments observed in ASD may be a reflection of deficiencies in executive control.

While the executive function and ToM link has been well-documented throughout the literature, less is known about its possible relationship with the affective domain of empathy. Some reports suggest a significant association between affective empathy and executive control processes in patients with frontotemporal dementia (Eslinger et al. 2011). However, there is a dearth of research examining this association in relation to ASD and typical development.

To summarise, in light of the emerging evidence suggesting a qualitatively similar (though milder) pattern of impairments among those with elevated levels of ASD traits, assessing autism symptomatology among typically developing populations is a promising way forward, potentially offering novel information about the social and non-social features of ASDs. A key advantage of examining typically developing individuals with ASD traits is that they are more likely to be tolerant of structured testing environments than those with a clinical diagnosis. Thus, we may be able to gain unique insights into the spectrum by employing a wider range of tasks and methodologies when studying this broader population.

Furthermore, establishing links between empathic functioning and executive control in ASD could have important implications for both clinical and non-clinical ASD. Specifically, a comprehensive examination of these processes could help identify key neurocognitive mechanisms that may influence the therapeutic efficacy of social interventions. Interventions within the interpersonal sphere typically focus on broader, more goal-oriented aspects of social interactions (improving general conversational skills, forming interpersonal relationships etc.) and their application to real-world settings. However, given the meaningful overlap between social and non-social domains of cognition, it may be necessary to remediate the deficits in more *‘basic’* neurocognitive processes, before targeting more higher-order social competencies. A multi-tier intervention strategy could, therefore, enhance positive outcomes and prove more effective in alleviating the negative consequences associated with social dysfunction in autism (e.g., peer-rejection, loneliness, and mental health difficulties; Bauminger and Kasari 2000; Chamberlain et al. 2007; Tantam 2003). Furthermore, given that the autism spectrum extends into the general population, typically developing individuals with elevated levels of ASD traits may also benefit from programmes supporting adaptive social functioning. However, a necessary prerequisite for devising such interventions is furthering our understanding of the neurocognitive processes associated with social dysfunction in ASD.

### The Present Study

The present two-study paper aimed to provide a comprehensive examination of the empathy, executive function, and ASD trait link in a sample of typically developing adults and adolescents. In study 1, we investigated multiple domains of empathic processing by utilising ecologically valid measures of cognitive (Movie for the Assessment of Social Cognition, MASC; Dziobek et al. 2006) and affective (Self-Assessment Manikin Faces Task, SAM; Seara-Cardoso et al. 2012) empathy. Our second study examined the association between empathic processing, ASD traits, and the executive domains of response inhibition, planning, and cognitive flexibility. Across both studies, we sought to determine the potential contribution of alexithymia to performance on measures of cognitive and affective empathy as well as to examine its association with multiple aspects of executive control.

Based on the evidence outlined above, we predicted that individuals with higher ASD traits would demonstrate poorer performance on measures of cognitive, but not affective, empathy (H1), and this impairment was expected to be more pronounced on the naturalistic MASC task relative to a static measure of cognitive empathy (i.e., Reading the Mind from the Eyes Test, EYES; Baron-Cohen et al. 2001a; H2). In addition to a unique contribution by subclinical autism traits, we expected a positive relationship between measures of executive control and the cognitive, but not the affective, domain of empathic processing (H3). We further hypothesised that individuals with higher levels of ASD traits would demonstrate poorer performance on tasks assessing executive control (H4).

With respect to alexithymia, higher levels of this trait have been shown to predict deficits in both cognitive (Moriguchi et al. 2006) and affective (Lockwood et al. 2013) empathy, along with impaired executive functioning (Koven and Thomas 2010). Consequently, we predicted that elevated levels of alexithymia would be related to poorer performance on measures of cognitive and affective empathy (H5) as well as on measures of executive control (H6). Consistent with the existing developmental literature reporting a protracted development of empathic and executive processes over the period of preadolescence and adulthood (Decety 2010; Dumontheil et al. 2010), we expected older participants to evidence better task performance on measures of cognitive empathy and executive control. In contrast, we did not expect to find a predictive relationship between age and the affective domain of empathy. Similarly, given that general cognitive ability has been associated with ToM performance and executive control abilities (Pellicano 2007), we incorporated IQ into our design as a control variable to examine whether ASD traits and executive function contribute unique variance to empathic processing over and above general cognitive ability.

## Study 1

### Method

#### Participants

One-hundred-and-twenty-four healthy adults and adolescents were recruited through a university subject pool and a London-based Sixth-Form college. Two participants were excluded from the analyses because they were missing data on one of the experimental tasks, and one because he or she scored above the clinical cut-off point (i.e., 32+) on the Autism Spectrum Quotient (AQ; Baron-Cohen et al. 2001c). This left a final sample of 121 participants (15 % male) aged 16-35 (*M* = 18.43, *SD* = 1.93), with IQs ranging between 72 and 129 (*M* = 102.02, *SD* = 11.55).

#### Measures

##### ASD Traits

The AQ (Baron-Cohen et al. 2001b) is a 50-item self-report questionnaire based on a 4-point Likert scale designed to assess autism traits in both clinical and community samples. Responses in the ‘autistic’ direction receive 1-point, whilst ‘non-autistic’ responses receive 0 points. Total scores range from 0 to 50, with higher scores indicating greater levels of autism symptomatology. Psychometric examination of the AQ has revealed good test–retest reliability and moderate-to-high internal consistency (Baron-Cohen et al. 2001c; Cronbach’s alpha was 0.67 in the present study), as well as good discriminative validity (Woodbury-Smith et al. 2005).

##### Alexithymic Traits

Alexithymic traits were assessed using the Toronto Alexithymia Scale (TAS; Bagby et al. 1994), a 20-item instrument comprising three dimensions: difficulty identifying feelings, difficulty describing feelings, and externally oriented thinking. Each item is responded to on a five-point Likert scale, ranging from “strongly disagree” to “strongly agree”. Total scores vary from 20 to 100, with higher scores indicating a greater degree of alexithymia. The TAS has generally shown robust psychometric properties (Bagby et al. 2007; Parker et al. 2003; Cronbach’s alpha was 0.81 in the present study).

### Assessment of Empathic Functioning

#### Cognitive Empathy

*Static Theory of Mind* The Reading the Mind from the Eyes Test (EYES; Baron-Cohen 2001a) is a widely used measure of theory of mind ability. It assesses the capacity to understand and infer the mental state of others from static images depicting the eye region of the face. Based on this visual information alone, respondents are required to choose which of four mental state terms (one target word and three foils) correctly depicts what the person in the picture is thinking or feeling. Two variants of this test were administered: the revised 36-item adult version (Baron-Cohen et al. 2001a; completed by all participants recruited via the subject pool) and the 28-item child version (completed by all participants recruited via a Sixth-Form college).

The adult EYES comprises complex mental state terms (e.g., ‘pensive’, ‘playful’, and ‘elated’), whilst the child version consists of simpler descriptors (e.g., ‘happy’, ‘sad’, and ‘scared’). Participants completing the adult version were informed that they could request an explanation of the descriptor meanings and could also consult a glossary, if they were unsure of any of the words used. One point was assigned for all correct answers and a percentage of accuracy score was calculated for each participant.

*Naturalistic Theory of Mind* The Movie for the Assessment of Social Cognition (MASC; Dziobek et al. 2006) is a naturalistic video-based mentalizing task that approximates the demands of real-life social situations. It involves watching a 15-min movie about four characters spending an evening together and answering questions concerning their mental states. The film incorporates themes about peer and romantic relationships, and requires participants to process information from visual (e.g., facial expressions and eye gaze), auditory (e.g., prosody), and verbal (e.g., content of language) channels. The film is paused at 45 points, and participants are asked to respond to questions relating to the characters’ thoughts, feelings, and intentions (e.g., “What is Cliff feeling?,” “What is Betty thinking?,” “Why is Michael doing this?”). Answer options are presented in a multi-choice format comprising four response options: (1) hypermentalizing (e.g., “she is exasperated about Michael coming on too strong”), (2) under/reduced mentalizing (e.g., “she is pleased about his compliment”), (3) no mentalizing (e.g., “her hair does not look that nice”), and (4) accurate mentalizing (e.g., “she is flattered but somewhat taken by surprise”). Accurate responses receive one point, and total scores vary between 0 and 45, with higher values indicating greater mentalizing ability. Adequate psychometric properties have been reported for the MASC (Dziobek et al. 2006; Lahera et al. 2014), with the task successfully distinguishing between healthy participants and individuals diagnosed with Asperger syndrome (Dziobek et al. 2006; Lahera et al. 2014), Schizophrenia (Montag et al. 2011), and borderline personality disorder (Preißler et al. 2010).

#### Affective Empathy

The Self-Assessment Manikin Faces Task (SAM) is an ecologically valid index of affective empathy (Lockwood et al. 2013; Seara-Cardoso et al. 2012). It requires participants to rate their own emotional response to pictures of faces displaying sad, fearful, angry, happy, or neutral expressions. Participants respond to each image using a 9-point valence scale, ranging from a low-spirited manikin (‘1’) to a cheerful one (‘9’), with a neutral manikin in the middle (‘5’). The sequence of images was randomized for each participant and the ratings for sadness, fear, and anger were reverse-scored so that higher scores reflected greater distress when viewing others’ negative emotions. These variables were subsequently transformed into z-scores and a composite score was created along with the ratings for happy expressions.

#### General Cognitive Ability

The full-scale IQ of each participant was measured using the two-subset form (Vocabulary and Matrix Reasoning) of the Wechsler Abbreviated Scale of Intelligence (WASI; Wechsler 1999).

#### Procedure

The study protocol was granted ethical approval from the university Research Ethics Committee, and written informed consent was obtained from all participants and from parents of adolescents. A series of tasks assessing social cognitive functioning were administered as part of a wider battery of measures. Each participant was tested individually for approximately two hours in a quiet, dimly lit, room. All tasks were presented in randomised order and instructions were provided at the beginning of each test. Participants were allowed to take short rest breaks between the tasks as needed. At the end of the test battery, an estimate of general intellectual functioning was obtained using the two-subtest form of the Wechsler Abbreviated Scale of Intelligence (Wechsler 1999).

### Results

The means, standard deviations, and bivariate correlation coefficients for all variables can be seen in Table 1. As hypothesized, ASD traits showed a significant negative correlation with MASC (*r* = −.404, *p* < .001) and EYES (*r* = −.218, *p* = .017) performance, but not with SAM scores (*r* = .023, *p* = .804). Alexithymia was negatively associated with MASC (*r* = −.402, *p* < .001) and EYES performance (*r* = −.303, *p* = .001), and positively associated with ASD traits (*r* = .437, *p* < .001). The negative association between TAS scores and SAM performance did not reach statistical significance (*r* = −.106, *p* = .249). Finally, whilst performance on the MASC and EYES tasks was positively correlated (*r* = .288, *p* = .001), neither of these ToM measures were significantly correlated with SAM performance (MASC: *r* = −.036, *p* = .692; EYES: *r* = .158, *p* = .083).

**Table 1:** Descriptive statistics and correlations between measures of autism spectrum disorder, alexithymic traits, and task performance

	M	SD	1	2	3	4
1. AQ	15.50	5.18				
2. TAS	46.83	10.04	.437**			
3. MASC	34.74	4.45	−.404**	−.402**		
4. EYES	79.34	10.10	−.218*	−.303**	.288**	
5. SAM	.001	1.73	.023	−.106	−.036	.158

*AQ* Autism Spectrum Quotient, *TAS* Toronto Alexithymia Scale, *MASC* Movie for the Assessment of Social Cognition, *EYES* Reading the Mind from the Eyes Test, *SAM* Self-Assessment Manikin Faces Task

* *p* < .05; ** *p* < .01

#### Hierarchical Regressions

We conducted hierarchical multiple regression in order to investigate the association between ASD traits and ToM performance, whilst controlling for alexithymia, age, and general cognitive ability. Beta estimates for the models are presented in Table 2. This analysis was intended to further assess the hypothesis that higher ASD traits would be related to greater difficulties in cognitive empathy (H1) and that and this impairment would be more pronounced on the MASC task relative to the EYES test (H2). For the model predicting naturalistic ToM performance, total MASC scores were first regressed onto Age and IQ scores. The regression model was significant (*R* = .43, *R*^2^ adj = .17, *F*_(2,118)_ = 12.99, *p* < .001), with the two predictors collectively explaining 18 % of the variance in MASC scores. Age (t_(118)_ = 2.28, *p* = .024) and IQ (t_(118)_ = 2.92, *p* = .004) were both positive predictors of MASC performance. At the second step alexithymia scores were entered. The regression model remained significant (*R* = .49, *R*^2^ adj = .22, *F*_(3,117)_ = 12.40, *p* < .001), with the three predictors jointly explaining 24 % of the variance in naturalistic ToM performance. Trait alexithymia (*t*_(117)_ = 3.06, *p* = .003) and IQ (*t*_(117)_ = 2.34, *p* = .021) were both uniquely associated with MASC performance, but Age (*t*_(117)_ = 1.48, *p* = .142) was no longer a significant predictor. The signs of the coefficients suggested that elevated levels of alexithymia and lower IQ scores were related to poorer MASC performance. The *R*^*2*^ change was significant (*F*_(1,117)_ change = 9.37, *p* = .003), indicating that including trait alexithymia explained significant additional variance in the model.

**Table 2:** Hierarchical regressions of naturalistic ToM and static ToM on IQ and age (Step 1), alexithymia (Step 2), and autism traits (Step 3)

	Naturalistic ToM task			Static ToM task		
	Beta	*t*	*p*	Beta	*t*	*p*
Step 1						
IQ	.277	2.919	.004**	.196	1.939	.055
Age	.216	2.283	.024*	.093	.920	.359
Step 2						
IQ	.218	2.335	.021*	.144	1.427	.156
Age	.140	1.477	.142	.025	.244	.808
TAS	−.272	3.061	.003**	−.244	2.534	.013*
Step 3						
IQ	.246	2.746	.007**	.155	1.531	.129
Age	.129	1.418	.159	.020	.200	.842
TAS	−.135	1.443	.152	−.190	1.800	.075
AQ	−.302	3.524	.001**	−.118	1.216	.227

*AQ* Autism Spectrum Quotient, *TAS* Toronto Alexithymia Scale. Full IQ calculated from the Wechsler Abbreviated Scales of Intelligence

* *p* < .05; ** *p* < .01

At the third and final step, ASD traits were entered. Once again, the regression model was significant (*R* = .56, *R*^2^ adj = .29, *F*_(4,116)_ = 13.31, *p* < .001), with the four predictors collectively explaining 32 % of the variance in MASC scores. Of the four predictors, only ASD traits (*t*_(116)_ = 3.52, *p* = .001) and IQ (*t*_(116)_ = 2.75, *p* = .007) were uniquely associated with naturalistic ToM performance. The signs of the coefficients indicated that elevated levels of ASD traits and lower IQ scores were related to difficulties in mental state attribution. The *R*^2^ change was significant (*F*_(1,116)_ change = 12.42, *p* = .001), suggesting that the inclusion of ASD traits explained significant additional variance in the model. Neither alexithymia (*t*_(116)_ = 1.44, *p* = .152), nor Age (*t*_(116)_ = 1.42, *p* = .159) reached significance levels as predictors in the final model.

To examine hypothesis H2, and to further test H1, the same regression sequence was applied to static ToM performance. At the first step, scores on the EYES test were regressed onto Age and IQ. The regression model was significant (*R* = .25, *R*^2^ adj = .05, *F*_(2,118)_ = 4.08, *p* = .019), with the two predictors explaining 7 % of the variance in EYES scores. Age (*t*_(118)_ = .920, *p* = .359) was a non-significant predictor of static ToM performance, whilst IQ (*t*_(118)_ = 1.94, *p* = .055) showed a trend towards significance. Trait alexithymia was entered at the second step. The regression model was significant (R = .34, *R*^*2*^ adj = .09, *F*_(3,117)_ = 4.98, *p* = .003), with the three predictors collectively explaining 11 % of the variance in EYES performance. Alexithymia (*t*_(117)_ = 2.53, *p* = .013) was uniquely and negatively associated with EYES performance, whilst Age (*t*_(117)_ = .224, *p* = .808) and IQ (*t*_(117)_ = 1.43, *p* = .156), did not reach significance. *R*^2^ change was significant (*F*_(1,117)_ change = 6.42, *p* = .013), indicating that the inclusion of trait alexithymia accounted for significant additional variance in the model.

 ASD traits were entered at the third step. Once again, the regression model was significant (R = .35, *R*^2^ adj = .09, *F*_(4,116)_ = 4.12, *p* = .004), with the four predictors collectively explaining 12 % of the variance in EYES scores. ASD traits (t_(116)_ = 1.22, *p* = .227), were not related to static ToM performance. The *R*^*2*^ change was not significant in this case (F_(1,116)_ change = 1.48, *p* = .227), suggesting that ASD traits did not explain additional variance in the model. Neither IQ (*t*_(116)_ = 1.53, *p* = .129), nor Age (*t*_(116)_ = .200, *p* = .842) reached statistical significance in this model. However, alexithymia showed a trend towards significance (*t*_(116)_ = 1.80, *p* = .075). Gender was included in the analyses and subsequently removed after returning non-significant results in all cases.

Overall, findings from our first study demonstrated that individuals with elevated levels of autism traits experience a similar pattern of difficulties in empathic processing as those with a clinical diagnosis of ASD. As hypothesized, findings showed that whilst mental state attribution is significantly impaired in those with higher levels of ASD traits, the ability to resonate with others’ emotions remains largely intact. Furthermore, findings from the hierarchical regressions suggested that individuals with higher ASD traits and lower IQ scores experienced greater difficulties in identifying mental states from dynamic video-based stimuli, but not from static images depicting the eye region of the face. However, despite significant associations with autism symptomatology and ToM ability, trait alexithymia did not explain the mentalizing difficulties in naturalistic ToM associated with elevated levels of ASD traits, and was not related to performance on the affective empathy task. Taken together, these findings yield strong support for H1 and H2, and provide partial support for H5.

## Study 2

### Method

#### Participants

One hundred and seven participants (16 % male) from the original sample returned to complete the second part of the research. Participants were aged 16–22 (*M* = 18.12, *SD* = 1.24), with IQs between 72 and 129 (*M* = 101.72, *SD* = 11.51). There were no statistically significant differences in IQ scores (*t*_(119)_ = .781, *p* = .437) between returning participants and those completing the first part only (*M* = 104.29, *SD* = 12.04).

#### Assessment of Executive Functioning

##### Cognitive Flexibility

A computerised set-shifting task (Smillie et al. 2009) based on the Wisconsin Card Sorting Test (WCST; Grant and Berg 1948), programmed in Matlab using the Psychophysics Toolbox extensions (Brainard 1997; Pelli 1997), was used to assess cognitive flexibility. Each trial entailed the presentation of a card, which varied in three different ways: (1) was blue or yellow in colour, (2) displayed either a ‘0’ or an ‘X’ on the front, and (3) appeared on the left or right side of the screen. Participants were instructed to sort the cards into two piles by pressing either the ‘\’ or ‘/’ key (which were marked as ‘A’ and ‘B’). At the end of each trial, participants were provided with feedback on the accuracy of their response.

Participants were told that to learn how to sort the cards correctly, they would need to use the accuracy feedback and learn by trial-and-error. After 10 consecutive correct responses, there was an unannounced switch in the sorting rule. A total of five shifts took place during the experiment, with each rule repeated twice. The task duration was approximately 10 min and finished once the participant had successfully completed all five shifts or once they had reached the maximum number of trials (120), whichever was earlier. Performance was assessed via the total number of shifts made and the shifting efficiency measure proposed by Cianchetti et al. (2005). This method of scoring awards six points for each shift that is successfully completed and a further point for each remaining trial, provided that all shifts are made before reaching 120 trials. For instance, a participant who has made all five shifts in 90 trials would receive a shifting efficiency score of 5 × 6 + (120–90) = 60.

##### Response Inhibition

The Go/No-Go (Form S3; Kaiser et al. 2010) is a widely used measure of response inhibition. In this task, the participants were required to respond as quickly and accurately as possible to all individually presented triangles (“go” trials), but to avoid responding to the circles (“no-go” trials). The stimuli were presented for 200 ms and the interstimulus intervals were 1000 ms. The Go/No-Go comprised two blocks and a total of 250 trials, 19 % of which were “no-go” trials and the remaining 81 % “go” trials. The main outcome variable recorded for this task was the number of trials in which the participant responded to a circle (Error of commission).

##### Planning Ability

A computerised version of the Tower of London (Freiburg Version, ToL-F; Kaller et al. 2012) task was used to assess planning ability. In this task, a set of differently coloured balls placed on three vertical rods of different heights are displayed on the screen. Participants are presented with a start state and instructed to re-configure the balls to match a given goal state while following three key rules: (1) Only one ball can be moved at a time, (2) balls cannot be placed outside the rods, and (3) if more than one ball is stacked on a rod, only the top ball can be moved. Participants were also instructed to solve each problem in the minimum number of moves set for each trial and to plan a solution before executing the sequence of movements.

Experimental trials were presented in order of ascending difficulty and comprised a total of 24 problems (eight four-move problems, eight five-move problems, and eight six move problems). The primary outcome measure for this task was number of problems correctly solved within a time limit of 1 min per trial (Planning ability).

#### Procedure

Participants were administered a series of executive function tasks in a quiet, dimly lit room. Task order was randomised across each session and participants were provided with instructions at the start of each test. The task-set took approximately 1.5 h to complete and participants were allowed to take short breaks between the tasks as needed. Once again, these data were collected as part of a wider battery of measures.

### Results

The means, standard deviations, and bivariate correlation coefficients for all variables are presented in Table 3. With the exception of WCST Efficiency scores and ToL-F performance (*r* = .295, *p* = .002), none of the executive function measures were interrelated (WCST Efficiency-Go/No-Go: *r* = −.057, *p* = .599; ToL-F-Go/No-Go: *r* = .112, *p* = .251). As predicted, MASC performance was positively associated with WCST Efficiency (*r* = .386, *p* < .001) and ToL-F (*r* = .247, *p* = .010) scores, and negatively associated with commission errors on the Go/No-Go (*r* = −.207, *p* = .032). Finally, none of the executive function measures were significantly associated with SAM performance (WCST Efficiency: *r* = .054, *p* = .578; ToL-F: *r* = −.034, *p* = .730; Go/No-Go: *r* = −.060, *p* = .524).

**Table 3:** Descriptive statistics and correlations between measures of empathic processing and executive control

	M	SD	1	2	3	4	5	6
1. MASC	34.92	4.35	–					
2. EYES	79.58	10.19	.284	–				
3. SAM	.01	1.74	−.090	.123	–			
4. WCST	25.02	16.00	.386**	.336**	.054	–		
5. GNG	13.41	6.31	−.207*	.016	−.060	−.057	–	
6. ToL-F	14.87	3.62	.247*	.194*	−.034	.295**	.112	–
7. Age	18.21	1.24	.407**	.001	−.001	.210*	−.122	.387**

*MASC* Movie for the Assessment of Social Cognition; *EYES* Reading the Mind from the Eyes Test; *SAM* Self-Assessment Manikin Faces task; *WCST* Wisconsin Card-Sort Test; *GNG* Go/No-Go; *ToL-F* Tower of London- Freiburg Version

* *p* < .05; ** p < *.01*

#### Hierarchical Regressions

We conducted hierarchical multiple regression analyses in order to investigate the association between ToM performance, executive functioning, and subclinical ASD traits whilst controlling for age and general cognitive ability. Beta estimates for the models are presented in Table 4. This analysis was intended to further assess the hypothesis that, in addition to a unique contribution by ASD traits, the cognitive, but not the affective, domain of empathy would be associated with executive control abilities (H3).

 For the model predicting naturalistic ToM performance, total MASC scores were first regressed onto Age and IQ scores. The regression model was significant (*R* = .45, *R*^2^ adj = .19, *F*_(2,104)_ = 13.25, *p* < .001), with the two predictors collectively explaining 20 % of the variance in MASC scores. Age (*t*_(104)_ = 2.79, *p* = .006) and IQ (*t*_(104)_ = 2.20, *p* = .030) were both positive predictors of MASC performance. At the second step ASD traits were entered. The regression model remained significant (*R* = .54, *R*^2^ adj = .27, *F*_(3,103)_ = 13.74, *p* < .001), with the three predictors jointly explaining 29 % of the variance in naturalistic ToM performance. ASD traits (*t*_(103)_ = 3.46, *p* = .001) were uniquely and negatively associated with MASC performance, whilst Age (*t*_(103)_ = 2.09, *p* = .039) and IQ (*t*_(103)_ = 2.53, *p* = .013) revealed unique positive associations. The *R*^*2*^ change was significant (*F*_(1,103)_ change = 11.94, *p* = .001), indicating that including ASD traits explained significant additional variance in the model.

**Table 4:** Hierarchical regressions of naturalistic ToM and Static ToM on IQ and age (Step 1), autism traits (Step 2), and executive control (Step 3)

	Naturalistic ToM task			Static ToM task		
	Beta	*t*	*p*	Beta	*t*	*p*
Step 1						
IQ	.226	2.197	.030*	.148	1.315	.191
Age	.288	2.794	.006**	.093	.829	.409
Step 2						
IQ	.249	2.532	.013*	.163	1.458	.148
Age	.211	2.094	.039*	.044	.384	.701
AQ	−.296	3.456	.001**	−.190	1.951	.054
Step 3						
IQ	.187	1.866	.065	.058	.503	.616
Age	.174	1.732	.086	.024	.213	.831
AQ	−.243	2.862	.005**	−.180	1.852	.067
WCST	.241	2.778	.007**	.272	2.749	.007**
Go/No-Go	−.127	1.505	.135	.068	.708	.481
ToL-F	.042	.447	.656	.070	.660	.511

*AQ* Autism Spectrum Quotient, *TAS* Toronto Alexithymia Scale, *WCST* Wisconsin Card-Sort Test, *GNG* Go/No-Go, *ToL-F* Tower of London- Freiburg Version. Full IQ calculated from the Wechsler Abbreviated Scales of Intelligence

* *p* < .05; ** *p* < .01

At the third stage, measures of executive function (i.e., WCST Efficiency, GNG, and ToL-F) were entered. The regression model was significant, (*R* = .60, *R*^2^ adj = .32, *F*_(6,100)_ = 9.30, *p* < .001), and together, the six predictors explained 36 % of the variance in MASC scores. ASD traits (*t*_(100)_ = 2.86, *p* = .005) and WCST performance (*β* = .24, *t*_(100)_ = 2.78, *p* = .007), emerged as significant predictors of naturalistic ToM performance. The signs of the coefficients suggested that higher ASD traits and lower levels of cognitive flexibility were related to difficulties in mental state attribution in a naturalistic context. The *R*^*2*^ change was significant (*F*_(3,100)_ change = 3.76, *p* = .013), suggesting that executive functioning explained significantly more variance in the model. None of the other predictors in the model reached statistical significance, Age (*t*_(100)_ = 1.73, *p* = .086), GNG (*t*_(100)_ = 1.51, *p* = .135), and ToL-F (*t*_(100)_ = .447, *p* = .656), although IQ (*t*_(100)_ = 1.87, *p* = .065) indicated a trend towards significance.

The same regression sequence was applied to static ToM performance. At the first step, scores on the EYES test were regressed onto Age and IQ. The regression model was non-significant, (*R* = .21, *R*^2^ adj = .03, *F*_(2,104)_ = 2.47, *p* = .089), and neither Age (*t*_(104)_ = .829, *p* = .409), nor IQ (*t*_(104)_ = 1.32, *p* = .191) reached individual statistical significance in the model. ASD traits were entered at the second step. The regression model was significant, (*R* = .28, *R*^2^ adj = .05 *F*_(3,103)_ = 2.96, *p* = .036). Collectively, the three predictors explained 8 % of the variance in EYES scores. The unique negative association between ASD traits (*t*_(103)_ = 1.95, *p* = .054) and EYES scores approached significance. The *R*^2^ change was significant at trend level (*F*_(1, 103)_ change = 3.81, *p* = .054), suggesting that ASD traits explain incremental variance in the model. Once again, Age (*t*_(103)_ = .384, *p* = .701) and IQ (*t*_(103)_ = 1.46, *p* = .148) did not reach statistical significance.

Measures of executive function (i.e., WCST Efficiency, GNG, and ToL-F) were entered at the third and final step. The regression model was significant, (*R* = .40, *R*^2^*adj* = .11 *F*_(6,100)_ = 3.17, *p* = .007), and together, the six predictors explained 16 % of the variance in EYES scores. WCST performance (*t*_(100)_ = 2.75, *p* = .007), emerged as a significant predictor of static ToM. The *R*^2^ change was significant (*F*_(3,100)_ change = 3.18, *p* = .027), indicating that including executive functioning explained significant additional variance in the model. None of the remaining predictors reached statistical significance: ASD traits (*t*_(100)_ = 1.85, *p* = .067), GNG (*t*_(100)_ = .708, *p* = .481), ToL-F (*t*_(100)_ = .660 *p* = .511), IQ (*t*_(100)_ = .503, *p* = .616), and Age (*t*_(100)_ = .213, *p* = .831).

Last, bivariate correlations were computed to assess the hypothesis that individuals with elevated levels of ASD traits (H4) and alexithymia (H6) would demonstrate poorer performance on tasks indexing executive control. Bivariate correlation coefficients for all variables are presented in Table 5. There was a significant positive correlation between ASD traits and commission errors on the GNG task (*r* = .250, *p* = .009). Analysis also revealed a significant negative association between ASD symptomatology and WCST Shift scores (*r* = −.224, *p* = .021). However, the negative correlations between ASD traits and WCST Efficiency scores and ToL-F performance were not statistically significant (*p* > .05). A similar pattern emerged with alexithymia. Whilst there was a significant positive association with GNG scores (*r* = .219, *p* = .023), the negative relationship with WCST (Efficiency: *r* = − .135, *p* = .165; Shift: *r* = −.134, *p* = .170) and ToL-F did not reach statistical significance (*r* = −.153, *p* = .116). Once again, gender was included in all analyses and subsequently eliminated after returning non-significant results.

**Table 5:** Descriptive statistics and correlations between measures of autism spectrum disorder, alexithymic traits, and executive control

	1	2	3	4	5
1. AQ	–				
2. TAS	.476**	–			
3. WCST Shift	−.224*	−.134	–		
4. WCST Efficiency	−.133	−.135	.829**	–	
5. GNG	.250**	.219*	−.152	−.057	–
6. ToL	−.013	−.153	.231*	.295**	.112

*AQ* Autism Spectrum Quotient, *TAS* Toronto Alexithymia Scale, *WCST* Wisconsin Card-Sort Test, *GNG* Go/No-Go, *ToL-F* Tower of London- Freiburg Version

* *p* < .05; ** *p* < .01

Taken together, findings from Study 2 indicated a substantial overlap between empathic processing, executive function, and ASD traits. Analysis revealed that higher scores on the naturalistic ToM task was associated with better performance across all components of executive processing, whilst static ToM was associated with planning and cognitive flexibility. By contrast, there were no statistically significant associations between the affective domain of empathy and executive function.

Our findings also demonstrated age-related improvements in naturalistic ToM as well as in the set-shifting and planning domains of executive control. However, the association between age, affective empathy, and commission errors on the response inhibition task did not reach statistical significance.

The hierarchical regressions suggested that accurately decoding mental states from video-based stimuli is associated with lower levels of autism symptomatology and flexible cognition. Of the executive function measures used in this study, accurate performance on the static EYES test was exclusively associated with set-shifting ability. In terms of the autism symptomatology and executive function relationship, findings showed that individuals with higher levels of ASD traits exhibit a profile of executive function impairments that is partially comparable to those reported in clinical ASD. Lastly, greater levels of alexithymia were also found to be associated with impaired response inhibition. However, the negative correlation between alexithymia and the executive domains of planning and set-shifting ability did not reach statistical significance. Overall, our data yielded strong support for H3, partial support for H4 and H6, and also indicate the existence of age-related advancements in mentalizing ability and executive control.

## Discussion

Recent investigations suggest that ToM and executive function are interrelated constructs following a protracted course of development, and that autism-related difficulties in social and executive processing extend beyond individuals diagnosed with ASD. Whilst there is increasing interest in the link between executive control and mentalizing, and in their respective relationships with autism, little relevant research has been conducted at the subclinical level. In the current study, we addressed this gap in the literature by examining the link between empathic functioning, executive control, and autism symptomatology in a sample of typically developing adults and adolescents. To our knowledge, this is the first study to provide a comprehensive investigation of these relationships in a non-clinical population. Our study replicates and extends previous work by showing that: (1) naturalistic ToM is linked to elevated levels of autism traits; (2) along with ASD traits, decoding mental states from dynamic stimuli is related to flexible cognition; (3) the positive association between autism traits and executive difficulties observed here partially parallels studies suggesting executive function deficits in clinical ASD; and (iv) impaired mentalizing ability in those with elevated levels of ASD traits is not explained by co-occurring alexithymia.

As hypothesized (H1 and H5), data from our first study showed that naturalistic ToM performance was negatively associated with autism symptomatology and trait alexithymia. By contrast, neither ASD traits nor alexithymia were related to performance on the affective empathy task. Whilst our findings in relation to impaired cognitive and spared affective empathy in ASD traits converge with existing autism literature (Dziobek et al. 2008; Jones et al. 2010; Lockwood et al. 2013; Schwenck et al. 2012), the lack of association between alexithymia and reduced affective empathy is somewhat surprising and inconsistent with previous reports (Bird et al. 2010; Lockwood et al. 2013). Nonetheless, the negative association between alexithymia and ToM performance yields support for previous work reporting alexithymia-related deficits in the cognitive domain of empathy (Moriguchi et al. 2006).

Our finding of a modest positive association between ASD traits and alexithymia replicated data reported in previous studies (Lockwood et al. 2013). In addition, the non-significant correlations between measures tapping cognitive and affective empathy suggest that these tasks capture distinct components of empathic processing. Further analysis revealed unique associations between ASD traits and impaired mentalizing ability on a naturalistic measure of ToM. While alexithymia and age did not make a significant contribution to task performance in this model, IQ emerged as an independent contributor to mentalizing ability on the naturalistic ToM task. In contrast, we did not observe any unique associations between ASD traits and performance on a static test of ToM (i.e., Reading the Mind from the Eyes Test; Baron-Cohen et al. 2001a). Taken together, these results suggest that individuals with higher levels of ASD traits and lower IQ experience significant difficulties in attributing mental states to movie characters in a real-life social context, but not to static images depicting the eye region of the face. This finding is of particular importance as it shows that, along with capturing more profound ToM deficits present in clinical populations (Dziobek et al. 2006; Lahera et al. 2014), the MASC is also sensitive in detecting subtle mindreading impairments in typically developing adults and adolescents.

Indeed, the fact that IQ made an independent contribution to naturalistic ToM performance suggests that along with autism symptomatology, general cognitive ability also plays a role in mental-state reasoning in typical development. This finding speaks against previous reports documenting non-significant associations between MASC performance and IQ scores (Dziobek et al. 2006; Lahera et al. 2014), and, instead, converges with other studies documenting a positive link between ecologically valid assessments of ToM and intellectual capacity (Heavey et al. 2000; Ponnet et al. 2008). However, it is worth noting that general cognitive ability was no longer significant once cognitive flexibility was incorporated into the model (Study 2). This suggests that flexible cognition accounts for the same variance in ToM as IQ, and is uniquely related to mentalizing ability. Together, these data indicate the involvement of multiple processes in successful mentalization, and highlight the value of incorporating non-social cognitive domains in studies of empathic processing.

In sum, Study 1 supported previous research documenting mentalizing deficits in typically developing individuals with higher levels of autism traits (Gökçen et al. 2014; Lockwood et al. 2013), and extended their findings to include a naturalistic measure of ToM. Interestingly, our results concerning alexithymia appear to be inconsistent with recent theory and evidence purporting that alexithymic traits account for the observed empathy deficits related to ASD (Bird and Cook 2013). Rather, our data are in line with Lockwood et al’s (2013), suggesting that alexithymia cannot explain the mindreading difficulties associated with elevated ASD traits.

Findings from our second study revealed significant associations between ToM performance and executive control. As expected, scores on a naturalistic measure of ToM were positively related with set-shifting and planning ability, and negatively associated with impaired response inhibition. A similar pattern emerged with static ToM performance, such that scores on this task were positively related to measures of cognitive flexibility and planning performance. Nonetheless, the correlation between static ToM ability and impaired response inhibition did not reach significance levels. Furthermore, none of the sub-domains of executive function were related to our measure of affective empathy. Age was also positively related to naturalistic ToM performance, as well as to the set-shifting and planning domains of executive function. However, the negative association between age and response inhibition failed to reach statistical significance. Interestingly, our results also indicate that although certain measures of executive control share some variance, they have the ability to capture different aspects of higher order processing.

Additional analyses revealed unique associations between ToM performance and executive control. For instance, along with ASD traits, cognitive flexibility also emerged as a significant predictor of naturalistic ToM. With regards to static ToM performance, findings showed that cognitive flexibility was the only predictor to reach statistical significance. Collectively, these findings suggest that autism symptomatology and flexible cognition are key factors associated with optimal performance on a naturalistic measure of cognitive empathy. However, of the social and executive function variables included in the present study, decoding mental states from static images appears to call solely upon the executive domain of cognitive flexibility.

The influential role of autism symptomatology and executive processing is perhaps not surprising given that the MASC provides a closer approximation of the intricacies involved in everyday social interactions. For example, by presenting dynamic interactions in a real-world context, this task empirically evaluates participants’ capacity to recognise characters’ thoughts, emotions, and intentions from multiple channels of communication. Since this measure provides a more complex and ecologically valid assessment of mentalizing ability, successful performance on this task is likely to be sub-served by key neurocognitive processes enabling flexible adaptation to changing social contexts, and the capacity to shift between our own and others’ perspectives during mental state reasoning. In contrast to video-based assessments of ToM, mental-state attribution to static images is likely to make less of a demand upon available processing resources.

It is worth noting that our data are inconsistent with previous work reporting non-significant associations between naturalistic ToM performance and executive control. One explanation for this apparent discrepancy may lie in the type of executive function tasks employed. For instance, Lahera et al. (2014) assessed the naturalistic ToM and executive control relationship using only a brief measure of neuro-cognition (i.e., Screen for Cognitive Impairment in Psychiatry; Purdon 2005; SCIP), rather than a comprehensive battery of tasks. In addition, Dziobek et al. (2006) administered a different set of experimental paradigms to assess executive functions (i.e., Stroop Test, Stroop 1935; Trail Making Test, Reitan and Wolfson 1993; and verbal fluency, Horn 1962), rendering a direct comparison with the present study considerably difficult. Therefore, replication of our methodologies and findings in ASD populations will help resolve the inconsistencies surrounding the association between naturalistic ToM and executive control.

Interestingly, the positive association between age and executive function, in combination with the finding that younger participants make more ToM errors, appears to be in line with the notion that social cognition and executive function follow a protracted developmental course (Decety 2010; Dumontheil et al. 2010). We also found that autism symptomatology was associated with increased executive problems. For instance, analysis showed that individuals with higher ASD traits evidenced poorer response inhibition and achieved fewer set-shifts on a measure of cognitive flexibility. However, it should be noted that the negative association between autism traits and shifting efficiency—a more sensitive index of flexible cognition—failed to reach statistical significance. In addition, no significant association was found between planning ability and ASD traits. With respect to alexithymia, impairments were only observed on the response inhibition domain of executive processing.

The observed relationships between autism symptomatology, cognitive flexibility, and impaired response inhibition are in line with previous reports from ASD populations (Pellicano 2007; Robinson et al. 2009; though see Hill 2004a, b), but partly contradict data from Christ et al’s (2010) study examining executive functioning in subclinical ASD traits. Again, the conflicting pattern of results should be viewed in light of the assessment techniques employed. For example, whilst Christ et al. (2010) used a self-report measure of higher-order processing, the present study administered a behavioral index of all relevant executive domains. Therefore, it is possible that the behavioral methodology employed by the current study is better able to detect individual differences in response inhibition. Together, these findings demonstrate that response inhibition and cognitive flexibility are adversely affected in those with higher levels of ASD traits, and suggest that the executive processing difficulties characterising ASDs extend beyond people with a clinical diagnosis, into the general population. However, given that people with clinical ASD typically exhibit deficits planning and cognitive flexibility, whilst response inhibition remains largely intact (Hill 2004a, b), these data yield only partial support for the hypothesis that individuals with high levels of subclinical autism traits have a similar profile of executive deficits as their peers with clinical ASD.

In addition to informing our understanding of the broader autism phenotype, these results also have implications for clinical practice. Importantly, they indicate that cognitive empathy and executive function are key processes to consider when designing intervention programmes targeting adaptive social functioning in typically developing populations with elevated levels of autism traits. With regards to clinical ASD, the finding that naturalistic ToM was related to deficits in cognitive flexibility suggests that this executive domain may be particularly relevant for enhancing the treatment effects of social interventions. In other words, a multi-tier approach to social interventions may be necessary to improve socio-adaptive outcomes and alleviate the direct and indirect negative consequences associated with interpersonal difficulties in ASD. A further implication of these findings concerns the selection of control participants in autism research. Controlling for ASD traits in typically developing populations may be particularly important when examining ToM and executive function abilities, as variability in these traits may influence task performance and hinder the accurate profiling of social and non-social processes in clinical ASD. The presence of significant group differences in social and executive processing abilities might, therefore, depend on whether control participants are nearer the higher or lower end of the broader autism spectrum range (von dem Hagen et al. 2011). Thus, establishing levels of subclinical autism symptomatology could provide a more accurate profile of the neurocognitive processes underpinning social dysfunction in ASD.

### Limitations

Most participants in this study were female, leading to a significant gender imbalance in our sample. This is a consequence of primarily recruiting Psychology students (undergraduate and A-level), where there is an evident female bias. Although analysis revealed no confounding by gender, the association between executive control and mentalizing difficulties may vary across males and females with elevated autism traits. For instance, recent investigations have reported gender-specific cognitive impairments in ASD, with high-functioning autistic males evidencing greater deficits in sub-domains of executive control relative to their female counterparts (Bölte et al. 2011; Lehnhardt et al. 2016). Thus, given that executive function impairments in ASD are partially modulated by sex, and that better executive control potentiates socio-communicative skills (Bölte et al. 2011), examining these processes in more balanced, or male-dominated samples could reveal a stronger association between executive control and ToM deficits. To address this gap in the literature, future investigations should compare male and female participants in order to help determine whether sex-related differences in neurocognitive processing extend to subclinical ASD. A further aim would be to ascertain the extent to which these differences influence the link between impaired ToM and executive dysfunction. As it stands, the pattern of results observed in the current study may be female-specific and limited in its generalizability to male samples. Second, the study does not address the issue of directionality between ToM and executive control deficits. The extent to which impairments in mentalizing ability may be accounted for by executive dysfunction is, therefore, unclear and warrants further investigation. Third, whilst naturalistic assessments provide a closer approximation of empathic processing, in real-life social situations, mental-state reasoning and empathic responses occur in the context of reciprocal social interactions. Consequently, it would be of particular interest to observe participants’ interpersonal competence in an experimental setting. Such a line of investigation would also help determine whether the processing deficits associated with elevated autism symptomatology can explain real-world social functioning. Finally, future investigations should also utilise dual-task paradigms to assess social and non-social information processing simultaneously. Whilst our data corroborate the notion that executive function and ToM are closely bound constructs, examining them in tandem could be instrumental to our understanding of successful social performance in everyday contexts and, ultimately, to the design of interventions programmes targeting interpersonal performance. More immediately, the potential importance for clinicians is highlighted of assessing both cognitive empathy and executive function in individualising programmes in order to support the social functioning of adolescents and young adults, even when there is no ASD diagnosis.

## Conclusions

In summary, the current research findings suggest that ASD traits, executive function, age, and general cognitive ability are important factors in optimal mentalizing ability. Moreover, they show that individuals with elevated levels of autism traits display a similar profile of difficulties in empathic functioning, and a partially comparable pattern of executive function deficits as those with a clinical diagnosis of ASD. Further investigation of these domains in both clinical and subclinical ASD has the potential to advance our understanding of the broader autism phenotype as well as to elucidate the neurocognitive underpinnings of adaptive social behavior.
